# Correction: Enhancing radiosensitivity of melanoma cells through very high dose rate pulses released by a plasma focus device

**DOI:** 10.1371/journal.pone.0204692

**Published:** 2018-09-21

**Authors:** The PLOS ONE Staff

There are errors in the author affiliations. The affiliations should appear as shown here:

Francesca Buontempo^1^, Ester Orsini^1^, Isabella Zironi^2,6,7^, Lorenzo Isolan^3,8^, AlessandraCappellini^4^, Stefania Rapino^5,7^, Agostino Tartari^3^, Domiziano Mostacci^3^, Giorgio Cucchi^3^, Alberto Maria Martelli^1^, Marco Sumini^3,6^, Gastone Castellani^2,6,7^

**1** University of Bologna, Department of Biomedical and Neuromotor Sciences, Bologna, Italy, **2** University of Bologna, Department of Physics and Astronomy, Bologna, Italy, **3** University of Bologna, Department of Industrial Engineering, Bologna, Italy, **4** University of Cassino and Southern Lazio, Department of Human Social and Health Sciences, Cassino, Italy, **5** University of Bologna, Department of Chemistry “G. Ciamician”, Bologna, Italy, **6** National Institute for Nuclear Physics (INFN), Bologna, Italy, **7** Interdepartmental Centre “L. Galvani” (CIG) for integrated studies of bioinformatics, biophysics and biocomplexity, Bologna, Italy, **8** European Institute of Oncology and Monzino Cardiac Center Foundation (IEO-CCM), Milano, Italy.

In the Funding section, the grant from the funder INFN ETHICS 2015 is incorrectly attributed to Dr. Giorgio Cucchi. The correct attribution is to Gastone Castellani. The publisher apologizes for the errors.
